# Endomorphin-2 Inhibition of Substance P Signaling within Lamina I of the Spinal Cord Is Impaired in Diabetic Neuropathic Pain Rats

**DOI:** 10.3389/fnmol.2016.00167

**Published:** 2017-01-10

**Authors:** Fa-Ping Wan, Yang Bai, Zhen-Zhen Kou, Ting Zhang, Hui Li, Ya-Yun Wang, Yun-Qing Li

**Affiliations:** ^1^Department of Anatomy and Histology and Embryology, K.K. Leung Brain Research Centre, The Fourth Military Medical UniversityXi’an, China; ^2^Collaborative Innovation Center for Brain Science, Fudan UniversityShanghai, China

**Keywords:** endomorphin-2, diabetic neuropathic pain, substance P, neurokinin-1 receptor internalization, μ-opioid receptor

## Abstract

Opiate analgesia in the spinal cord is impaired in diabetic neuropathic pain (DNP), but until now the reason is unknown. We hypothesized that it resulted from a decreased inhibition of substance P (SP) signaling within the dorsal horn of the spinal cord. To investigate this possibility, we evaluated the effects of endomorphin-2 (EM2), an endogenous ligand of the μ-opioid receptor (MOR), on SP release within lamina I of the spinal dorsal horn (SDH) in rats with DNP. We established the DNP rat model and compared the analgesic efficacy of EM2 between inflammation pain and DNP rat models. Behavioral results suggested that the analgesic efficacy of EM2 was compromised in the condition of painful diabetic neuropathy. Then, we measured presynaptic SP release induced by different stimulating modalities via neurokinin-1 receptor (NK1R) internalization. Although there was no significant change in basal and evoked SP release between control and DNP rats, EM2 failed to inhibit SP release by noxious mechanical and thermal stimuli in DNP but not in control and inflammation pain model. We also observed that EM2 decreased the number of FOS-positive neurons within lamina I of the SDH but did not change the amount of FOS/NK1R double-labeled neurons. Finally, we identified a remarkable decrease in MORs within the primary afferent fibers and dorsal root ganglion (DRG) neurons by Western blot (WB) and immunohistochemistry (IHC). Taken together, these data suggest that reduced presynaptic MOR expression might account for the loss of the inhibitory effect of EM2 on SP signaling, which might be one of the neurobiological foundations for decreased opioid efficacy in the treatment of DNP.

## Introduction

Diabetic neuropathic pain (DNP) is one of the most prevalent complications of diabetes mellitus (DM) and takes a heavy toll on physical health and life well-being in DM patients. DNP management remains a major challenge due to its resistance to traditional opioids (Mousa et al., 2013; Shaqura et al., 2013). Although patients suffering from DNP could gain pain relief from high-dose opioids (Harati et al., 2000; Watson et al., 2003), the unbearable side-effects, such as sedation, respiratory depression, tolerance and opioid-induced hyperalgesia (OIH; Grider and Ackerman, 2008; Zhao et al., 2010), hampered its clinical application.

Endomorphin-2 (EM2), an endogenous μ-opioid receptor (MOR) ligand, has shown remarkable analgesic properties and fewer adverse effects. EM2 overcomes pain by activating pre- and postsynaptic MORs (Wu et al., 2003; Leng et al., 2005; Fujita and Kumamoto, 2006; Fichna et al., 2007; Chen Y. B. et al., 2015) along the spinal nociceptive transmission pathway. Spinal administration of EM2 has been demonstrated to alleviate nociceptive behaviors in inflammatory pain (Tateyama et al., 2002; Zhao et al., 2015), cancer pain (Chen L. et al., 2015) and peripheral neuropathic pain models (Przewlocka et al., 1999). However, there are few reports concerning the analgesic effects of EM2 at the spinal level in the DNP model.

Presynaptic MORs play an important role in opioid analgesia (Kohno et al., 2005; Chen and Pan, 2006). Opioids inhibit the release of excitatory neurotransmitters; specifically, the inhibition of substance P (SP) release is one of the presynaptic mechanisms for opioid analgesia (Kondo et al., 2005; Chen et al., 2014). In the neuropathic pain model of chronic constriction injury (CCI), the loss of inhibition of opioids on SP release within the spinal dorsal horn (SDH) contributed to the low analgesic efficacy of opioids (Chen et al., 2014), which was probably owing to the dysfunctional signaling mechanisms of presynaptic MORs (Hervera et al., 2011). Additionally, previous data has suggested that the level of MORs in dorsal root ganglion (DRG) neurons and the SDH were decreased in DNP rats (Chen et al., 2002; Mousa et al., 2013; Shaqura et al., 2013, 2014), which might account for reduced opioid analgesic efficacy. Considering this, we proposed that the decrease in the inhibitory effects on presynaptic SP release by opioids might be a potential link between reduced presynaptic MOR expression and decreased opioid analgesia in the condition of painful diabetic neuropathy.

Accumulating evidence has suggested that endogenous SP release can be evoked by noxious stimuli. SP released from primary afferent terminals activates the neurokinin 1 receptor (NK1R) in the spinal cord and leads to NK1R internalization (Trafton et al., 1999, 2001; Trafton and Basbaum, 2000; Kondo et al., 2005; Chen et al., 2014). In this study, the effects of EM2 on SP signaling were examined by two postsynaptic measures of SP release: NK1R internalization to reflect the amount of functional presynaptic SP release and FOS expression to examine the activity of NK1R postsynaptic neurons, as was adopted in previous studies (Trafton et al., 1999; Riley et al., 2001). In addition, lamina I of the SDH was selected as the observation zone based on following reasons: first, both NK1R-like immunoreactive (NK1R-LI) neurons and SP-like immunoreactive (SP-LI) fibers are densely encountered in lamina I (Littlewood et al., 1995). Second, NK1R internalization induced by acute noxious stimuli have been observed mainly in lamina I (Mantyh et al., 1995; Abbadie et al., 1997; Allen et al., 1997; Riley et al., 2001). Third, nearly 70% of lamina I neurons projecting to the parabrachial nucleus express NK1R (Ding et al., 1995).

The present study aimed at exploring the underlying mechanisms of decreased analgesic efficacy of EM2 in DNP disease. Firstly, basal and evoked SP release in control and DNP rats were compared. Then, the effects of EM2 on noxious stimulation-induced presynaptic SP release and FOS expression of postsynaptic NK1R-positive neurons, that is, the SP signaling within lamina I of the SDH, in DNP rats were examined. Finally, Western blot (WB) and immunohistochemistry (IHC) were combined to measure the expression of MOR in DRG neurons and primary afferent terminals in the SDH.

## Materials and Methods

### Experimental Animals

Male *Sprague-Dawley* rats weighing 200–220 g were used in this study. Before experiments, all animals were adapted to the experimental circumstances for 5–7 days. All procedures were approved by the Institutional Animal Care and Use Committee of the Fourth Military Medical University (Xi’an, P.R. China). Every measure was taken to minimize the number of animals and alleviate their discomfort. For the DM model, rats were given a single intraperitoneal injection of streptozotocin (STZ; 60 mg/kg; Sigma-Aldrich, St. Louis, MO, USA) dissolved in 0.1 M ice-cold citrate buffer (pH 4.5). On the third day, blood glucose values were measured with a glucometer (Accu-Chek Active, Roche, Basel, Switzerland). Rats with random blood glucose values higher than 16.7 mmol/L were further used. Age-matched vehicle rats were used as the control group. For the complete Freund’s adjuvant (CFA) inflammation model, 100 μl of CFA (Sigma-Aldrich, St. Louis, MO, USA) was injected into the subcutaneous surface of the left hindpaw. On the third day, rats with obvious pathological pain were selected for later behavioral and morphological studies.

### Pain Behavioral Test

All the behavioral tests were performed between 9:00 am and 6:00 pm. Before behavioral testing, animals were habituated in the testing apparatus for at least 30 min until they calmed down. For mechanical allodynia, von Frey filaments were applied with increased forces from 0.4 g to 60.0 g to test the paw withdrawal threshold (PWT; Morrow, 2004; Cui et al., 2014). Briefly, the von Frey hairs were pressed vertically on the hind plantar surface for approximately 4–5 s; each filament was used ten times; and a 5-min interval was left between the different forces. The minimal force that caused lifting or licking responses at least five times was considered as the PWT. Thermal hyperalgesia was tested by the Hargreaves method (Hargreaves et al., 1988; Wu et al., 2014). The infrared heat was applied to the hind plantar surface to induce paw withdrawal. The time from initiation of the light beam to paw withdrawal was recorded from an automated device readout as paw withdrawal latency (PWL). The intensity of the beam was set to produce a basal PWL of approximately 14–16 s, and a cut-off time of 35 s was set to prevent excessive tissue damage. Each foot was tested four times, and a 10-min interval was left before the next test.

### Intrathecal Implantation and Drug Administration

The lumbar catheterization was performed as previously described (Størkson et al., 1996; Chen et al., 2014). Briefly, under isoflurane (4%) anesthesia, a midline incision was made to expose the intervertebral space between L5 and L6. After a clear exposure, a polyethylene-10 catheter (0.28 mm i.d. and 0.61 mm o.d., Becton Dickinson, Sparks, MD, USA) was inserted into the subarachnoid space and pushed rostrally to terminate at the level of L4–L5 spinal segments. Then the PE-10 tube was tunneled under the skin and the incisions were sutured. Three days after catheterization, 10 μl of lidocaine (2%; followed by 10 μl saline for flushing) was injected into the catheter to test the successful insertion. Rats with signs of bilateral hind limbs paralysis immediately after lidocaine injection were selected for further experiments. All rats were housed separately to recover for 5–7 days. The following drugs were administered in this study, EM2 (No. E3148; Sigma-Aldrich), SP (No. 1156; Tocris) and L-732138, (NK1R antagonist, No. 0868; Tocris). The drugs were dissolved in sterile saline and administered intrathecally in a volume of 10 μl solution followed by 10 μl saline for flushing.

### Hindpaw Noxious Stimulation

Both noxious mechanical and thermal stimulation were employed in this study. The noxious mechanical stimulus (MS) was applied by clamping the left hindpaw with a hemostat for 30 s in 4% isoflurane anesthesia. In terms of nociceptive thermal stimulation, the left hindpaw (below the ankle joint) was immersed in 50°C water for 1 min. After stimulation, anesthesia was stopped and the rats were allowed to wake. No additional interventions were administrated to the rats until they were sacrificed. To examine noxious stimulation-induced NK1R internalization, the rats were perfused 10 min after the stimulation. To observe double labeling of NK1R and FOS, the rats were sacrificed 90 min after the stimulation. For the experiments that evaluated the effects of EM2 on SP release, EM2 was injected 10 min before the stimulation.

### Immunofluorescent Staining

Rats were deeply anesthetized with 2% sodium pentobarbital (60 mg/kg, *i.p.*). When the pain reflex disappeared, 200 ml of 0.01 M phosphate buffer saline (PBS) were perfused in the ascending aorta, followed by 500 ml of ice-cold fixative (0.1 M phosphate buffer (PB) containing 4% (w/v) paraformaldehyde, pH 7.2–7.4). After post-fixation in the same solution for 2–4 h, the lumbar segments of the spinal cord and DRG (L4–L5) were removed and placed in a 30% (w/v) sucrose solution for 24 h at 4°C. The sagittal serial sections (30 μm in thickness for L4–L5 spinal segments) and horizontal sections (20 μm in thickness for the DRG) were cut using a freezing microtome (Leica CM1950; Heidelberg, Germany) and collected into 0.01 M PBS (floating sections). After incubating in a blocking solution (10% (v/v) normal donkey serum) for 1 h at room temperature (RT), the sections were incubated with the following antibodies in sequence (antibodies shown in Table 1): (1) primary antibodies for 48 h (for single and double staining) or 72 h (for triple staining) at 4°C; (2) secondary antibodies for 4–6 h at RT; and (3) FITC-labeled avidin D (for single and double staining) or Alexa Fluor 594-labeled avidin D (for triple staining) for 2 h at RT. Some other sections were used as controls by replacing the primary antibodies with the combinations of normal serum according to the species of the primary antibodies used, while keeping the other conditions unchanged. Between the steps, the slices were rinsed with 0.01 M PBS for 30 min. After all steps, the slices were mounted onto glass slides, coverslipped with 50% glycerol, and conserved at 4°C.

**Table 1:** **Antibodies used in immunofluorescent staining**.

	Antigens	Primary antibodies	Secondary antibodies	Tertiary antibodies
Single staining	NK1R or	rabbit anti-NK1R antibody (1:200) (S8305; Sigma-Aldrich, St. Louis, MO, USA)	biotinylated donkey anti-rabbit IgG (1:500) (AP182F; Millipore, Billerica, MA, USA)	FITC-labeled avidin D (1:1000) (A-2001; Vector, Burlingame, CA, USA)
	MOR	rabbit anti-MOR antibody (1:500) (ab10275; abcam, Cambridge, MA, USA)		Alexa Fluor 594- avidin D (1:1000) (S32356; Invitrogen, Carlsbad, CA, USA)
Double staining	NK1R and	rabbit anti-NK1R antibody (1:200) (S8305; Sigma-Aldrich, St. Louis, MO, USA)	biotinylated donkey anti-rabbit IgG (1:500) (AP182F; Millipore, Billerica, MA, USA)	FITC-labeled avidin D (1:1000) (A-2001; Vector, Burlingame, CA, USA)
	FOS	mouse anti-c-Fos antibody (1:500) (ab11959; abcam, Cambridge, MA, USA)	Alexa Fluor 594 donkey anti-mouse IgG (1:500) (A-21203, Invitrogen, Carlsbad, CA, USA)	
Triple stainning	MOR and	rabbit anti-MOR antibody (1:500) (ab10275; abcam, Cambridge, MA, USA)	biotinylated donkey anti-rabbit IgG (1:500) (AP182F; Millipore, Billerica, MA, USA)	Alexa Fluor 594- avidin D (1:1000) (S32356; Invitrogen, Carlsbad, CA, USA)	
	SP and	guinea pig anti-Substance P antibody (1:200) (ab10353; abcam, Cambridge, MA, USA)	Alexa Fluor 488 donkey anti-guinea pig IgG (1:500) (706-545-148; Jackson West Grove, PA, USA)	
	NeuN	mouse anti-NeuN antibody (1:500) (NG1715199; Millipore, Billerica, MA, USA)	Alexa Fluor 647 donkey anti-mouse IgG (1:500) (A31571; Invitrogen, Carlsbad, CA, USA)	

### Quantification of Immunoreactivity

The amount of NK1R internalization was quantified as previously described (Abbadie et al., 1997; Trafton et al., 1999, 2001; Riley et al., 2001). In lamina I of the SDH, the NK1R-positive neurons that included the nucleus and a large area of cytoplasm were selected. NK1R-positive neurons with 10 or more endosomes in the soma were considered as internalized neurons (Zhang et al., 2013). The ability of NK1R internalization was represented as the proportion of NK1R-internalized cells (NK1R-internalized cells/NK1R-positive cells). Neurons from four sagittal sections were counted from each rat, and there were four rats in each group.

Quantification of FOS and NK1R double-labeling was performed according to previous studies (Trafton et al., 1999; Riley et al., 2001). The number of FOS-positive cells, NK1R-positive cells and NK1R/FOS double-labeled cells within lamina I of the SDH were counted independently. Counts were obtained from four sagittal sections per rat and presented as the average numbers per 300 μm sagittal section.

For quantitative analysis of triple labeling in the DRG, we counted positive cell bodies of SP, MOR and SP/MOR-expressing neurons. Only the neurons crossing nuclei were selected to count (Cui et al., 2014). The percentages of single-labeled neurons out of NeuN-positive neurons and that of double-labeled neurons out of MOR-like immunoreactivity (MOR-LI) or SP-LI neurons were calculated. Four sections were obtained from each rat. There were four rats in each group. Optical density of MOR-LI within the SDH was measured using the software “Image-Pro Plus 6.0”.

### Confocal Images

All confocal images were taken with a confocal laser scanning microscope (FV1000; Olympus, Tokyo, Japan). For NK1R labeling, all images were taken from sagittal sections of L4–L5 spinal segments and generated by superimposition of three optical sections. For MOR labeling, the images were taken from horizontal sections of the DRG or coronal sections of the spinal cord. Images were processed using Adobe Photoshop CS2 to adjust the contrast.

### Western Blotting

Rats were deeply anesthetized with 2% pentobarbital (60 mg/kg, *i.p.*). The dorsal part of the L4–L5 spinal segments and L4–L5 DRG were removed immediately and homogenized in lysis buffer containing proteinase inhibitors and phosphatase inhibitors (Roche, Switzerland). The protein concentration was tested using a bicinchoninic acid (BCA) assay (Pierce, Rockford, IL, USA). Proteins were separated by polyacrylamide gel electrophoresis. The antibodies used were as follows: rabbit-anti-MOR antibody (1:1000; ab10275; Abcam), mouse anti-β-actin antibody (1:5000; A1978; Sigma-Aldrich), peroxidase-conjugated goat anti-rabbit IgG (1:5000; AP132P; Millipore), and peroxidase-conjugated goat anti-mouse IgG (1:5000; AP124P; Millipore). The protein bands were visualized with an enhanced chemiluminescence (ECL) kit (Pierce, Rockford, IL, USA) and detected by the ChemiDoc Imaging System (Bio-Rad, Berkeley).

### Data Analysis

All the data were presented as the mean ± SEM. Data was performed using GraphPad Prism 5.01 (San Diego, CA, USA) for plotting and analysis. Statistical analyses were conducted using two-way ANOVA and Bonferroni’s post-test for treatment condition (control vs. drug) for analyzing the effect of EM2 on pain behaviors, NK1R internalization and FOS expression in NK1R-positive neurons. Student’s *t*-test was used for analyzing the data in the experiments for the establishment of the DM model and comparing the expression of MORs or SP in the DRG and SDH. *P* < 0.05 was considered statistically significant.

## Results

### Establishment of the Diabetes Mellitus Rat Model

**Figure 1:**
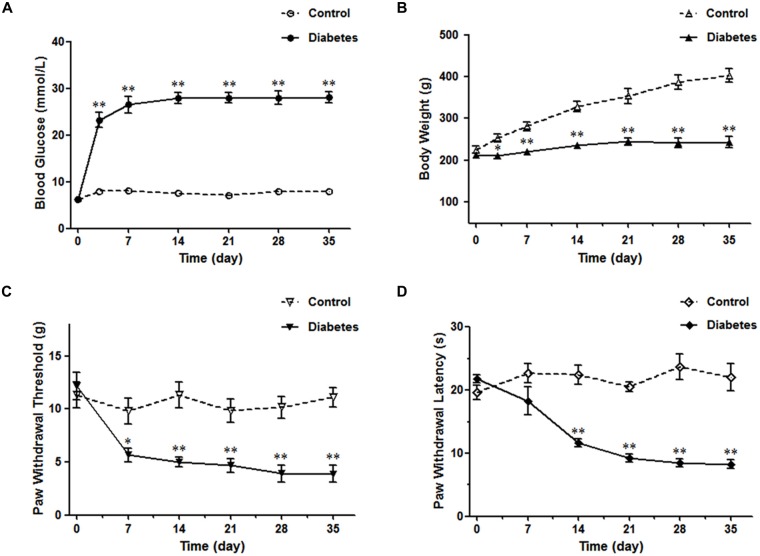
**Diabetes mellitus (DM) rat model induced by streptozotocin injection.** The diabetic rats exhibited high blood glucose values **(A)**, reduced body weights **(B)**, decreased paw withdrawal threshold (PWT) **(C)** and paw withdrawal latency (PWL) **(D)**. *n* = 6/group. *p < 0.05, **p < 0.01 vs. vehicle-control rats.

STZ-induced diabetic rat is the most commonly used model in experimental diabetes research (Morrow, 2004). The blood glucose reached the diabetic level (>16.7 mmol/L, 300 mg/dl) 3 days after STZ injection and was sustained for at least 5 weeks (Figure 1A). Meanwhile, the body weight did not increase, further ensuring the establishment of the DM model (Figure 1B). After the onset of DM, five time points (the 7th, 14th, 21st, 28th and 35th day) were selected to test the PWT and PWL. The diabetic rats began to show obvious mechanical sensitivity 7 days after STZ injection, which continued until the 35th d of DM (Figure 1C). In parallel, a dramatic thermal hyperalgesia to noxious plantar heat stimulation was observed in diabetic rats (Figure 1D). Subsequent experiments were performed 4 weeks after the STZ injection.

### EM2 Exhibited a Lower Antinociceptive Efficacy in the DNP Rats

**Figure 2:**
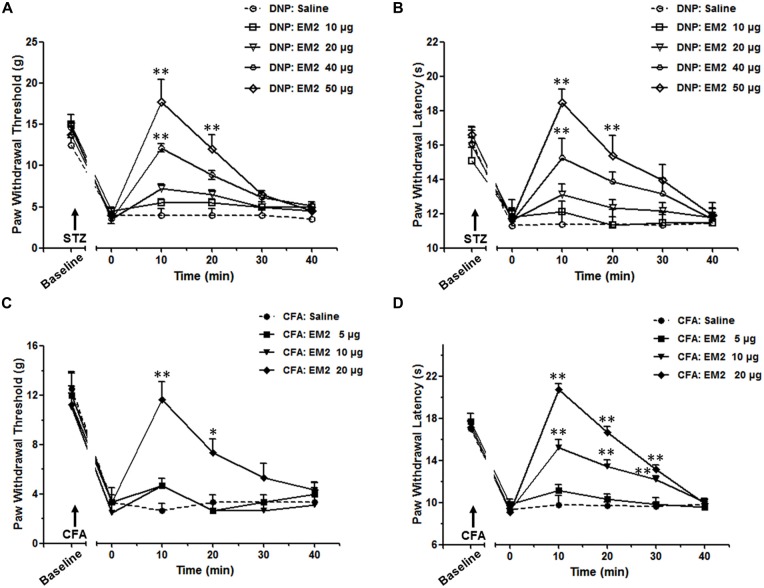
**The analgesic effects of endomorphin-2 (EM2) on mechanical allodynia and thermal hyperalgesia were impaired in the diabetic neuropathic pain (DNP) rats.** On the 28th day of DM, intrathecal injection of EM2 exhibited dose-dependent analgesic effects on both mechanical allodynia **(A)** and thermal hyperalgesia **(B)** in the DNP rats. The most potent effect was observed 10 min after injection and lasted for 10–20 min. Three days after complete Freund’s adjuvant (CFA) injection, EM2 dose-dependently decreased the PWT **(C)** and PWL **(D)** in the inflammation rats. The most potent dose was 50 μg and 20 μg in the DNP and CFA rats, respectively. *n* = 4/group. *p < 0.05, **p < 0.01, vs. saline-injected rats.

According to prior studies (Przewlocka et al., 1999; Przewlocki et al., 1999), a 2-fold increase in dose is adopted in our study, which could reveal the different analgesic effects of EM2 in these two pain models. The data showed intrathecal (*i.t.*) delivery of EM2 exhibited dose-dependent analgesic effects on both the mechanical allodynia and thermal hyperalgesia of the DNP rats (Figures 2A,B). In rats with mechanical allodynia, the potent analgesic dose of EM2 was 50 μg instead of 10 μg or 20 μg, which reached its peak at the 10 min (*P* < 0.05), lasted for at least 10 min and disappeared 30 min after EM2 injection (Figure 2A). Similarly, 50 μg was the minimum dose of EM2 that eliminated thermal hyperalgesia (Figure 2B). In addition, tests were conducted in the CFA inflammatory pain model as the control; EM2 showed a similarly potent antinociceptive effect, but the potent analgesic dose was 20 μg (Figures 2C,D). Taken together, EM2 had a transient, dose-dependent analgesic effect in both DNP and CFA rats, but the analgesic effects of EM2 were impaired in the DNP model.

### EM2 Inhibition of SP Release Was Hampered in the DNP Rats

#### Basal and Evoked NK1R Internalization in the DNP Rats were Identical to the Control Rats

**Figure 3:**
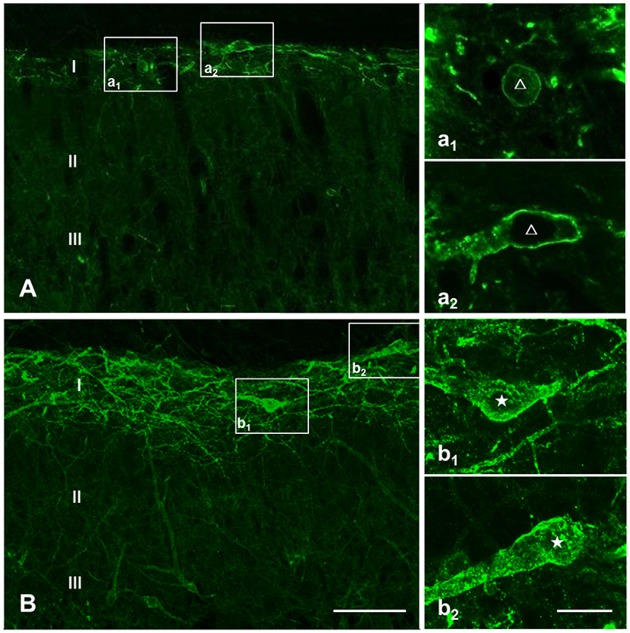
**Basal and substance P (SP)-evoked neurokinin-1 receptor (NK1R) internalization within the spinal dorsal horn (SDH).** Confocal images illustrate the dorsoventral pattern of NK1R-like immunoreactivity (NK1R-LI) within the SDH in a naive **(A)** and a SP-treated **(B)** rat. NK1R-LI was distributed on the membrane of the soma and dendrites in lamina I (i.e., “non-internalized”; **A, a_1_, a_2_**). NK1R-LI was present as bright, immunofluorescent endosomes in the cytoplasm of the soma and dendrites (i.e., “internalized”; **B, b_1_, b_2_**) throughout the dorsal horn, and was most prominent in lamina I. NK1R non-internalized and internalized neurons are indicated with a “△” and “★”, respectively. Scale bar = 100 μm **(A,B)**, 20 μm **(a_1_–b_2_)**.

**Figure 4:**
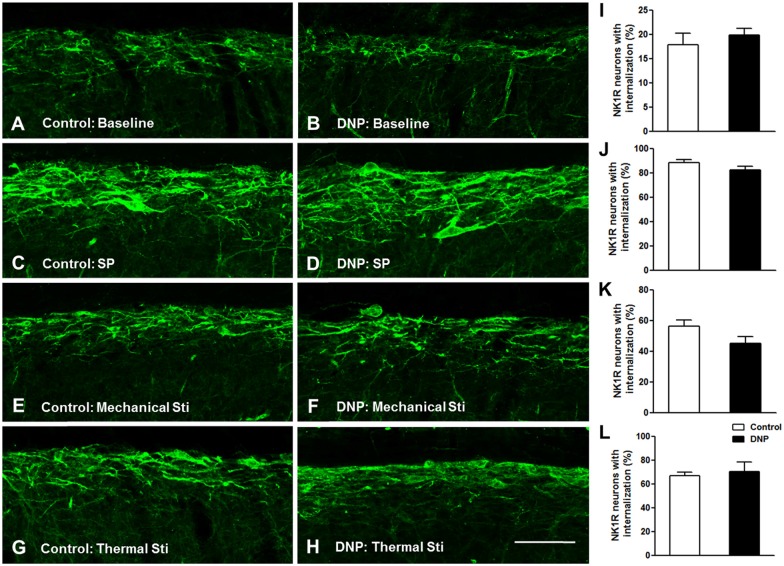
**No significant changes in basal and evoked NK1R internalization between the control and DNP rats.** The confocal images illustrate basal **(A,B)** and evoked **(C–H)** NK1R internalization within superficial lamina of the SDH by different stimulation modalities. **(A,B)** Non-stimulus; **(C,D)** SP treatment (*i.t.*, 30 nM); **(E,F)**, 30 s clamping; **(G,H)**, hot stimulus (50°C, 1 min). Statistical analysis for NK1R-LI showed that there were no differences in basal **(I)** and evoked NK1R **(J–L)** internalization between the two groups. *n* = 4/group. Scale bar = 100 μm.

Prior to clarifying the effects of EM2 on SP release from primary afferent terminals in the DNP rats, we tested the responses of NK1Rs to different stimulation modalities. Our results illustrated that the pattern of NK1R-LI was similar to that described previously (Figure 3; Littlewood et al., 1995; Abbadie et al., 1997). In rats without stimulation, most of the NK1R-LI was found on the membrane of somas and dendrites (i.e., “non-internalized”) in lamina I, and only a small fraction exhibited signs of internalization (Figures 4A,B). There was no significant difference in the control and DNP groups (17.85 ± 2.09% vs. 19.8 ± 1.2%; *F* = 3.019, *P* = 0.521; Figure 4I). Numerous NK1R-internalized cells were observed in the two groups (88.4 ± 2.14% vs. 82.5 ± 2.61%; *F* = 1.489, *P* = 0.204) 10 min after SP injection (Figures 4C,D,J). After clamping hindpaws for 30 s, although there was an obvious decline in the ratio of NK1R internalization in DNP rats, there was no significant difference between the two groups (56.3 ± 3.35% vs. 45.1 ± 3.8%; *F* = 1.287, *P* = 0.128; Figures 4E,F,K). The same situation occurred in the two groups of rats treated with noxious thermal stimuli (66.86 ± 2.78% vs. 69.1 ± 6.43%; *F* = 6.201, *P* = 0.734; Figures 4G,H,L). All of these results implied that there was no significant change in basal and evoked SP signaling activity between control and DNP rats with the different stimulation modalities.

#### EM2 Inhibition of SP Release was Impaired in the DNP Rats

**Figure 5:**
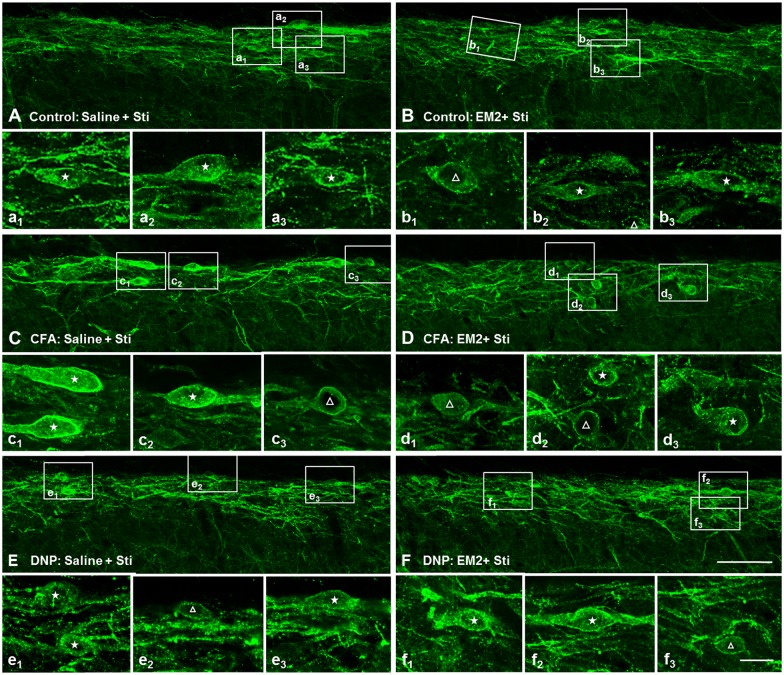
**The inhibitory effect of EM2 on NK1R internalization evoked by thermal stimuli was impaired in the DNP rats.** The confocal images of NK1R labeling in lamina I illustrate the changes in NK1R internalization after EM2 injection (*i.t.*) in the control (POD 28 days; **A,B**), CFA (POD 3 days; **C,D**) and DNP (POD 28 days; **E,F**) rats. There was a decrease in number of NK1R-internalized cells in control **(B)** and CFA **(D)** but not in DNP **(F)** rats after EM2 administration. Saline injection (10 μl; **A,C,E**). EM2 injection (50 μg; **B,F**). EM2 injection (20 μg; **D**). NK1R non-internalized and internalized neurons are indicated with a “△” and “★”, respectively. Scale bar = 100 μm (shown in **D** for **A–D**), 20 μm (shown in **f_3_** for **a_1_–f_3_**).

**Figure 6:**
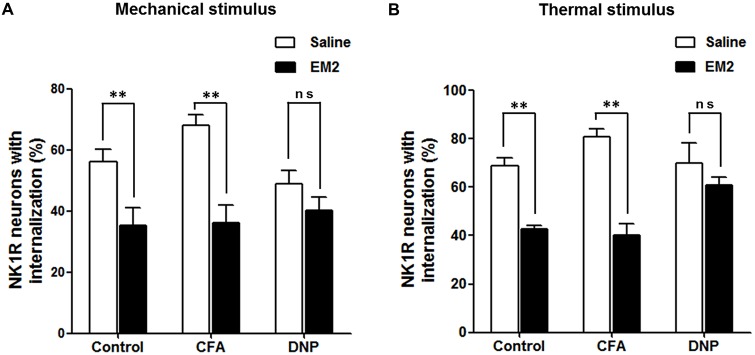
**EM2 decreased the level of NK1R internalization evoked by noxious stimulation in the DNP but not in the non-diabetic rats.** The bar graphs show that a potent analgesic dose of EM2 decreased the numbers of NK1R-internalized cells in the control and CFA but not DNP rats under the condition of both mechanical **(A)** and thermal **(B)** stimuli. 4–6 slices from a rat. *n* = 4–5/group. **p < 0.01.

To determine the role of SP signaling during the EM2 analgesic process, we observed the effects of a potent dose of EM2 on NK1R internalization induced by noxious stimulation. Since previous data suggested that opioids suppressed SP release effectively in inflammation pain (Chen et al., 2014), we evaluated NK1R internalization in CFA rats as a parallel control group. The results illustrated that EM2 significantly decreased the proportion of NK1R internalization induced by noxious thermal stimulation in vehicle-control (66.86 ± 2.78% vs. 42.85 ± 1.15%; *F* = 5.888, *P* = 0.009; Figures 5A,B, 6B) and CFA rats (80.88 ± 3.32% vs. 40.28 ± 4.61%; *F* = 1.932, *P* = 0.006; Figures 5C,D, 6B). However, in the DNP rats, this inhibitory influence of EM2 on SP release disappeared (69.1 ± 6.43% vs. 60.7 ± 2.94%, *F* = 5.561, *P* = 0.393; Figures 5E,F, 6B). Similar tendency of changes of NK1R internalization appeared in these three groups of rats treated with noxious mechanical stimulation (Figure 6A). The results demonstrated that the inhibitory effects of EM2 on NK1R internalization induced by both of the two noxious stimulation were impaired in the DNP but not in the control and CFA rats.

#### EM2 was Unable to Affect Exogenous SP-Induced NK1R Internalization

**Figure 7:**
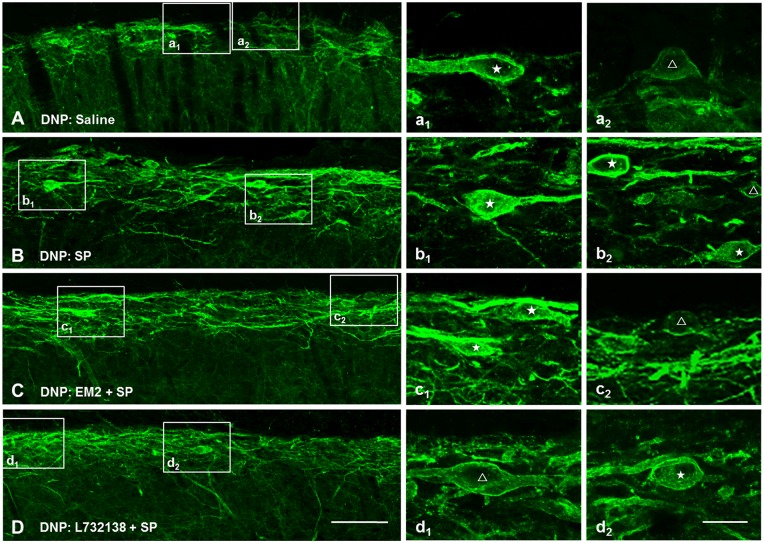
**EM2 had no direct influence on exogenous SP-induced NK1R internalization.** Representative confocal images illustrate that exogenous SP-induced NK1R internalization was inhibited by pretreatment with L-732138 instead of EM2 in the DNP rats. **(A)** 10 μl saline; **(B)** 30 nM SP (*i.t.*); **(C)** pretreatment with 50 μg EM2 (*i.t.*) 10 min before 30 nM SP (*i.t.*); **(D)** pretreatment with 100 nM L-732138 (*i.t.*) 10 min before 30 nM SP (*i.t.*). NK1R non-internalized and internalized neurons are indicated with a “△” and “★”, respectively. *n* = 4/group. Scale bar = 100 μm (shown in **D** for **A–D**), 20 μm (shown in **d_2_** for **a_1_–d_2_**).

NK1R internalization not only provides a measure of presynaptic SP release, but also provides a functional measure of SP signaling (Trafton et al., 1999; Riley et al., 2001). To test whether EM2 directly affects the internalization of NK1R, we observed the effect of EM2 on NK1R internalization induced by exogenous SP in DNP rats. The present results showed that compared to saline-injection rats (Figure 7A), SP (*i.t.*, 30 nM) induced a significant NK1R internalization within the superficial layers of the SDH (82.5 ± 2.61%, Figure 7B), which was not affected by injection of EM2 (*i.t.*, 50 μg, 10 min before SP injection; 81.62 ± 2.86%, *F* = 1.201, *P* = 0.828; Figure 7C) but was decreased by L732138 (*i.t.*, 100 nM, 10 min before SP injection; Cahill and Coderre, 2002; Zhang et al., 2013; Figure 7D). These results indicated that EM2 had no direct effects on NK1R internalization.

### EM2 Failed to Reduce FOS Expression in the NK1R-Positive Neurons in DNP Rats

**Figure 8:**
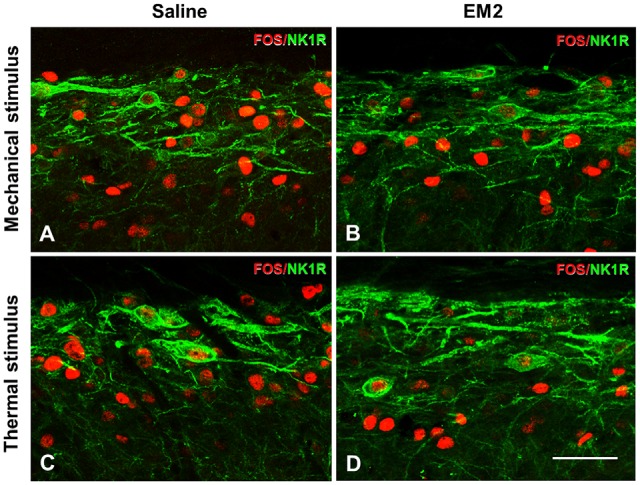
**EM2 decreased FOS-LI but not NK1R-LI and FOS/NK1R double-labeled neurons within the SDH in the DNP rats.** The NK1R (green) and FOS (red) double-staining images illustrated that EM2 decreased FOS-expressing neurons but not FOS/NK1R double-labeled neurons within lamina I of the SDH. **(A,C)** Pretreatment with 10 μl saline; **(B,D)** pretreatment with 50 μg EM2. *n* = 3–4/group. Scale bars = 50 μm.

A combination of FOS and NK1R staining was further used to test the effects of EM2 on NK1R-positive neurons. The results illustrated that in the DNP rats after mechanical stimulation, approximately 70% of the NK1R-LI cells also expressed FOS, while only about 10% of Fos-LI neurons were NK1R-positive neurons (Figure 8A, Table 2). EM2 reduced the number of FOS-expressing neurons in the SDH but not that of NK1R-positive and NK1R-FOS double-labeled cells (Figure 8B, Table 2). The same situation occurred in the DNP rats after thermal stimulation (Figures 8C,D, Table 2).

**Table 2:** **Effects of endomorphin-2 (EM2) on FOS/NK1R cells in the diabetic neuropathic pain (DNP) rats (*n* = 3–4)**.

Groups	FOS-positive cells	NK1R-positive cells	FOS in NK1R-positive cells
M S + saline	36.64 ± 1.86	5.14 ± 0.59	3.77 ± 0.58
M S + EM2	20.32 ± 2.54	5.02 ± 1.02	3.45 ± 1.14
T S + saline	39.72 ± 5.91	5.25 ± 1.54	4.26 ± 1.25
T S + EM2	19.95 ± 4.36	5.38 ± 1.13	4.15 ± 1.53

*MS, mechanical stimulus; TS, thermal stimulus*.

### DNP Reduced the Expression of MORs in the Primary Sensory Neurons and Their Central Terminals

**Figure 9:**
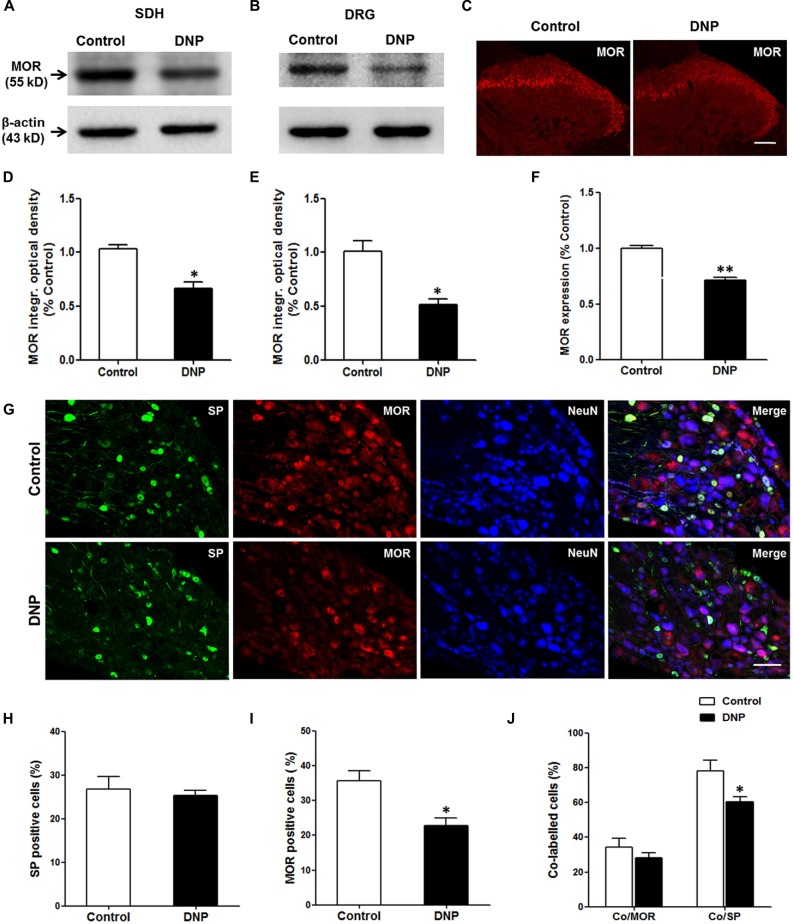
**Reduced expression of μ-opioid receptors (MORs) in neurons of the dorsal root ganglion (DRG) and SDH in the DNP rats.** Western blot (WB) bands illustrated that the level of MORs was decreased in the SDH **(A)** and DRG **(B)** of the DNP rats. Statistical analysis of the level of MORs within the SDH **(D)** and DRG **(E)**. Immunohistochemistry (IHC) results showed that there was a decrease of MOR labeling within the SDH in the DNP rats **(C)**. Statistical analysis of the expression of MORs within the SDH **(F)**. Triple labeling of SP (green), MOR (red) and NeuN (blue) in the DRG slices showed that the expression of MOR-like immunoreactivity (MOR-LI) but not SP-like immunoreactive (SP-LI) neurons in the DRG was reduced in the DNP rats. Meanwhile, the expression of MORs in SP-positive cells was also reduced **(G)**. Quantitative analysis of the numbers of SP-LI neurons **(H)**, MOR-LI neurons **(I)** and SP/MOR double-labeled neurons **(J)** in the DRG of the control and DNP rats. Four slices from each rat were used for immunofluorescent staining, *n* = 4/group. *p < 0.05, **p < 0.01, vs. vehicle-control rats. Scale bar = 100 μm.

MORs localized in primary central terminals mediates EM2 analgesia in the spinal cord. The loss of EM2 inhibition of SP release in DNP might be caused by the decrease in MOR expression in the DRG neurons. To test whether this is the case, we measured the level of MOR proteins in the DGR and spinal cord. The WB analysis illustrated that the expression of MOR proteins in DNP rats decreased to 66.8 ± 5.82% in the spinal cord (Figures 9A,D) and 51.77 ± 5.18% in the DRG (Figures 9B,E) of that in the control. This result was further confirmed by MOR labeling in the SDH (71.77 ± 2.42% to control; Figures 9C,F) and DRG (35.68 ± 2.84% vs. 22.75 ± 2.27%, *F* = 1.579, *P* = 0.012; Figures 9G,I). To reveal the change in MORs in the SP-LI neurons, we then performed triple staining for MOR, SP and NeuN in the DRG. The results illustrated that there was no difference in the proportion of SP-LI cells between the control and DNP rats (Figures 9G,H). However, the expression of MOR-LI in SP-ergic neurons was reduced in DNP rats (78.26 ± 6.09% vs. 60.57 ± 2.66%, *F* = 5.242, *P* = 0.037; Figure 9J).

## Discussion

### Spinal EM2 Analgesia in the DNP Rats

Together with EM-1, EM-2 (Tyr-Pro-Phe-Phe-NH2) is considered as an endogenous MOR ligand since it was one of the first peptides isolated from brain binding to MOR with high affinity and selectivity (Zadina et al., 1997). EM2-ir is primarily restricted to the superficial lamina of the SDH, where it plays the analgesic role in suppressing pathological pain (Sakurada et al., 1999, 2001). EM2 could be an ideal substitute for traditional morphine-like opioids owing to its remarkable antalgic properties and less adverse effects than opioids (Czapla et al., 2000; Varamini et al., 2013). What’s more, the mechanisms underlying EM2 analgesia may be more complicated. Bearing similarity to opioids, EM2 activates both pre- and postsynaptic MORs, leading to presynaptic inhibition of excitatory neurotransmitters (glutamate, SP and CGRP) release (Soldo and Moises, 1998; Leng et al., 2005; Heinke et al., 2011) and postsynaptic hyperpolarization of excitatory interneurons (Wu et al., 2003; Fujita and Kumamoto, 2006; Chen Y. B. et al., 2015), respectively. Interestingly, EM2 renders a specific binding site for SP1–7 (Botros et al., 2006, 2008; Fransson et al., 2010), a major bioactive metabolite of SP, and may mediate the antinociceptive effects of SP1–7 in pathological pain (Jonsson et al., 2015). More importantly, EM2 and morphine dissimilarly modulated MOR mRNA expression and function (McConalogue et al., 1999; Yu et al., 2003). Recently, pharmacologically modified endomorphin analogs aiming at enhancing its membrane permeability and resilience to enzymatic degradation have been developed which are prospective to proceed from research to the pharmaceutical industry (Varamini and Toth, 2013; Janecka and Gentilucci, 2014).

Our previous study suggested that the decreased expression of EM2 and MOR in the spinal cord was related to the progress of painful diabetic neuropathy (Kou et al., 2016). In the present study, we evaluated the analgesic efficacy of exogenous EM2 in DNP. Our behavioral testing showed that EM2 alleviated both mechanical and thermal hyperalgesia in the DNP and CFA rats. Concerning the antinociceptive effects of EM2 in inflammatory pain, it has been reported that EM2 dose-dependently increased the tail-flick latency in formalin-induced pain and a dose of 10 μg was enough to generate analgesic effects (Przewlocka et al., 1999). However, the potent dose of EM2 was higher (20 μg) in our experiment, which may result from the discrepancy in pain model (acute or chronic inflammatory pain) and behavioral measurement (tail flick test or von Frey filaments; Przewlocka et al., 1999). Importantly, comparing to the CFA model, the DNP rats required a significantly higher dose of EM2 to exhibit analgesic effects, suggesting that EM2 analgesic efficacy is impaired. Opioid resistance is a common phenomenon in the treatment of DNP. In patients with diabetic neuropathy, high doses of opioids (tramadol and oxycodone) were needed to gain equivalent pain-relieving effects (Harati et al., 2000; Watson et al., 2003). In rodent models of STZ-induced diabetic neuropathy, the antinociceptive efficacy of opioids after systemic (Courteix et al., 1998), spinal (Chen and Pan, 2003; Mousa et al., 2013; Shaqura et al., 2013) or supraspinal (Zurek et al., 2001) administration is reduced relative to controls.

### No Changes in Basal and Evoked SP Signaling in the DNP Rats

The neuropeptide SP, one of the most important pain mediators, is synthesized in small-sized DRG neurons and contributes to the development of chronic pain by activating the NK1R (Mantyh et al., 1997; Cahill and Coderre, 2002; Marvizón et al., 2003). In the present study, we examined the change in basal and evoked NK1R internalization within lamina I in the condition of DNP. In rats without stimulation, there was no significant change in NK1R internalization in DNP rats compared to the control, suggesting that the baseline SP release from peptidergic fibers was not changed. This was consistent with microdialysis data showing that the basal level of SP in spinal cerebrospinal fluid was not different in diabetic and control rats (Calcutt et al., 2000). Similarly, in rats with both mechanical and thermal stimuli, there were no significant changes in the amounts of internalized neurons between the two groups, indicating that evoked presynaptic SP release from the peptidergic fibers was not changed, either. However, striking discrepancies existed among our data and prior studies. Paw formalin injection attenuated SP-LI levels in spinal cord dialysates (Calcutt et al., 2000), while* in vitro* K^+^ administration evoked an excessive release of SP from the spinal cord in diabetic rats (Kamei et al., 1991). These differences might be derived from the heterogeneity of these studies (*in vitro* or *in vivo*, stimulation modality and detective method). However, our results were in agreement with another study suggesting that dorsal root stimulation failed to influence NK1R internalization in spinal cord slices of CCI rats (Chen et al., 2014).

The fact that there was no significant change in basal and evoked presynaptic SP release suggested that the SP signaling within lamina I of the SDH was not sensitized in the condition of diabetic neuropathy. Our previous study has reported the increased presynaptic glutamate release within the SDH in DNP rats (Kou et al., 2016). Therefore, SP signaling might not play an essential role in the central sensitization of painful diabetic neuropathy and this might account for the inefficacy of NK1R antagonists in the treatment of DNP (Sindrup et al., 2006).

### SP-NK1R Signaling Was Insusceptible to Opioid Analgesia in the DNP Rats

The main objective of the present study was to investigate the inhibitory influence of EM2 on SP release in the DNP rats. The results illustrated that the analgesic dose of EM2 failed to reduce the number of NK1R-internalized neurons induced by noxious stimulation in DNP rats but not in control and CFA rats. Similarly, a loss of the inhibition of SP release by DAMGO has been reported in the CCI model (Chen et al., 2014). Considering that opioids had better analgesic effects in CFA and control rats than in DNP and neuropathic pain rats (Przewlocka et al., 1999; Mousa et al., 2013; Shaqura et al., 2013; Zhang et al., 2016), the loss of inhibition of SP release may contribute to opioid resistance in chronic neuropathic pain.

A combination of FOS and NK1R labeling is a good way to evaluate the activity of SP-NK1R signaling (Trafton et al., 1999; Riley et al., 2001). In DNP rats, EM2 reduced FOS expression in the SDH but not in NK1R-positive neurons, indicating that the SP nociceptive pathway was insusceptible to EM2 analgesia in the DNP rats. Pretreatment with an NK1R antagonist but not EM2 blocked exogenous SP-induced NK1R internalization. This phenomenon, together with the unchanged number of FOS/NK1R double-labeled neurons after EM2 administration in DNP rats, implied that EM2 had no direct effect on NK1R-internalized neurons. The fact that there was no expression of MORs in NK1R neurons (Spike et al., 2002; Song and Marvizon, 2003) also excluded the possibility of a postsynaptic mechanism of EM2 to inhibit SP signaling.

Previous studies have provided possible mechanisms underlying the incapability of opioids in suppressing presynaptic SP release. Neurokinin release from central terminals is controlled by numerous presynaptic modulatory receptors, including inhibition by GABA_B_ (Marvizón et al., 1999; Riley et al., 2001; Zhang et al., 2010), α_2_-adrenergic (Takano et al., 1993) and opioid (Kondo et al., 2005; Beaudry et al., 2011; Chen et al., 2014) receptors, and facilitation by capsaicin (Marvizón et al., 2003), serotonin (Inoue et al., 1997) and NMDA (Liu et al., 1997; Trafton and Basbaum, 2000) receptors. In the condition of DNP, the overexpression of NMDA receptors (Tomiyama et al., 2005; Rondon et al., 2010) and decreased function of GABA_B_ (Wang et al., 2007) at the primary afferent terminals mobilizes excessive SP release (Marvizón et al., 1999) and counteracts the effects of EM2. In the present study, we provided evidence that the decreased level of presynaptic MORs might account for the reduced efficacy of EM2 on SP release.

### Down-Regulation of MORs Contributes to Lower Opioid Analgesic Efficacy

The MOR in primary terminals plays an important role in opioid analgesia (Kohno et al., 2005; Chen and Pan, 2006). Indeed, the down regulation of MORs has been found in many opioid-resistant neuropathological pain models (Chen et al., 2002; Cahill et al., 2003; Kohno et al., 2005; Mousa et al., 2013; Shaqura et al., 2013, 2014). The present study demonstrated reduced expression of MOR-LI fibers within the SDH, which was probably owing to the loss of MOR/SP double-staining sensory neurons. Here, a hypothesis for the effects of exogenous EM2 on SP signaling within the SDH in painful diabetic neuropathy was proposed: compared to the normal condition, SP-NK1R signaling within lamina I was not sensitized in the case of DNP during nociceptive message transmission; In addition, SP-NK1R signaling activated by noxious stimuli was insusceptible to exogenous EM2, probably owing to the loss of presynaptic MORs in the condition of diabetic neuropathy.

Sensory neuron apoptosis might not be a primary cause of the loss of MOR-LI terminals since there was no change in the total number of DRG neurons in STZ-treated rats (Zochodne et al., 2001). Instead, MOR displacement from the membrane to perinuclear compartments was responsible for the loss of presynaptic MOR expression within the SDH (Mousa et al., 2013). In addition to the loss of MOR expression, the impairment of MOR-G protein coupling and the reduction in G protein subunits have also been reported to be correlated with morphine insensitivity in DNP rats (Chen and Pan, 2003; Hajializadeh et al., 2010). These changes also occurred in injury-induced neuropathic pain (Zhang et al., 1998). However, in inflammatory pain, up-regulated MOR expression on peripheral sensory neurons potentiated opioid antinociception (Mousa et al., 2007). Thus, the variety of MOR signaling function determines the analgesic efficacy of opioids in different types of pathological pain.

Apart from the loss of control of SP release, decreased presynaptic MOR expression could also lead to decreased opioid efficacy via other mechanisms. Our previous study indicated that decreased presynaptic MOR expression led to attenuated inhibition on presynaptic glutamate release by EM2 during STZ-induced diabetes (Kou et al., 2016). Moreover, a loss in functional MORs on DRG neurons was a contributing factor to the impaired opioid inhibitory effects on capsaicin-induced TRPV1 activity in DNP rats (Shaqura et al., 2014). Considering this, current measures concerning the promotion of MOR signaling provide a promising orientation to facilitate opioid analgesia (Cahill et al., 2003; Mousa et al., 2007, 2013; Shaqura et al., 2013). Importantly, long-term EM2 administration in the early stages of DNP partially recovered the diminished spinal MOR expression and ameliorated painful diabetic neuropathy (Kou et al., 2016). Therefore, although a single dose of EM2 as the present study showed, exhibited lower analgesic effects, long-term EM2 pharmacotherapy may be a hopeful strategy in the field of clinical DNP management.

In addition to neural mechanisms, glial factors also contributed to reduced opioid efficacy as well as its side effects, namely, OT and OIH. On one hand, excessive inflammatory factors released by hyperactive microglia in the condition of neuropathic pain (Ji et al., 2013) may impede opioid efficacy since microglia inhibitors could increase morphine analgesia (Posillico et al., 2015; Castany et al., 2016); on the other hand, opioids activate microglia and astrocytes which in turn aggravates pain, and interrupting neuron-glia interaction could prevent tolerance and OIH (Mika, 2008; Grace et al., 2016; Roeckel et al., 2016). In light of these, a combination of endomorphin and glial inhibitors may be a novel and promising method for the treatment of neuropathic pain.

## Conclusion

In summary, our findings showed that SP-NK1R signaling is refractory to EM2 analgesia in STZ-induced diabetic rats. The diminished inhibition of EM2 might be attributed to reduced expression of presynaptic MORs. This might account for the impaired analgesic efficacy of opioids in the treatment DNP.

## Author Contributions

F-PW, YB and Z-ZK contributed equally to this manuscript as first authors. Y-QL designed the experiments. F-PW, Z-ZK and TZ performed the experiments. F-PW, YB and Y-QL wrote the manuscript. Y-YW and HL revised the manuscript. All authors read and approved the final version of the manuscript.

## Conflict of Interest Statement

The authors declare that the research was conducted in the absence of any commercial or financial relationships that could be construed as a potential conflict of interest.
